# Longitudinal Associations Between Sources of Uncertainty and Mental Health Amongst Resettled Refugees During the COVID-19 Pandemic

**DOI:** 10.3390/ijerph22060855

**Published:** 2025-05-30

**Authors:** Belinda J. Liddell, Stephanie Murphy, Yulisha Byrow, Meaghan O’Donnell, Vicki Mau, Tadgh McMahon, Richard A. Bryant, Philippa Specker, Angela Nickerson

**Affiliations:** 1 School of Psychological Sciences, University of Newcastle, University Drive, Callaghan, NSW 2308, Australia; 2 School of Psychology, University of New South Wales, Sydney, NSW 2052, Australiay.byrow@unsw.edu.au (Y.B.); r.bryant@unsw.edu.au (R.A.B.); p.specker@psy.unsw.edu.au (P.S.); a.nickerson@unsw.edu.au (A.N.); 3 Phoenix Australia, University of Melbourne, Carlton, VIC 3053, Australia; mod@unimelb.edu.au; 4 Australian Red Cross, Docklands, VIC 3008, Australia; 5 Settlement Services International (SSI), Ashfield and College of Public Health and Medicine, Flinders University, Bedford Park, SA 5042, Australia; tmcmahon@ssi.org.au

**Keywords:** refugee, COVID-19, trauma, PTSD, depression, intolerance of uncertainty, family separation, visa insecurity, longitudinal

## Abstract

The COVID-19 pandemic may have disproportionately affected forcibly displaced people due to parallel uncertainties such as visa insecurity and family separation. This study explicitly examined whether different sources of uncertainty contributed in specific ways to increased psychological symptoms for refugees during the pandemic. A large cohort of 733 refugees and asylum seekers settled in Australia completed a mental health survey in June 2020 (T1) and 12 months later in June 2021 (T2). Using cross-lagged panel modelling, we tested changes in post-traumatic stress (PTS), depression and anxiety symptoms, visa status, family separation and COVID-19 uncertainty stress, and the contribution of intolerance of uncertainty (trait prospective and inhibitory), controlling for age, sex, trauma exposure, language, and time in Australia. Visa status and family separation stress at T1 predicted increased depression (bidirectional pathways) and PTS symptoms at T2 (unidirectional pathways), respectively. Visa status uncertainty at T1 was also associated with increases in COVID-19 and family separation stress at T2. Intolerance of uncertainty showed limited associations with symptoms and stressors. Findings demonstrate that different forms of refugee uncertainty had specific impacts on psychopathology during the first year of the COVID-19 pandemic. Refugees facing diverse kinds of stress may benefit from individual, community, and policy level support targeted to their specific circumstances and mental health needs during future crises.

## 1. Introduction

Refugees are forcibly displaced over international borders and are unable to return to their home countries due to threat, violence, or persecution. Despite being protected under international law, refugees can face considerable hardship post-displacement with many living in states of protracted uncertainty regarding their future. For example, many high-income destination countries including the United Kingdom, United States, and Australia impose restrictive immigration policies on refugees and asylum seekers who seek safety but arrive irregularly, i.e., without a valid visa [1]. These policies can include mandatory immigration detention, relocation to an off-shore location while claims for asylum are being processed, or being provided with temporary visas with restricted access to services, family reunification or permanent resettlement pathways [2,3]. Visa insecurity and family separation are therefore significant forms of uncertainty experienced by refugees, and are both independently associated with adverse psychological outcomes [4,5,6,7,8]. When the COVID-19 pandemic emerged in 2020, there was international concern that refugees would be placed under additional strain given their prior trauma and exposure to uncertainty in relation to visa and family status, and therefore be particularly vulnerable to the adverse effects of the uncertainties inherent in a global pandemic [9,10,11]. For instance, it was observed that being reminded of traumatic events of the past due to COVID-19 public health measures (e.g., lockdowns) was a prominent predictor of PTSD, depression, anxiety, and health anxiety amongst resettled refugees in Australia [12]. Yet no study has examined the relative contribution of different forms of uncertainties commonly experienced by refugees on psychological outcomes during a time of global uncertainty, i.e., the COVID-19 pandemic. As such, this study sought to understand the specific mental health effects of different sources of refugee uncertainty, i.e., visa insecurity, family separation and COVID-19-related uncertainties on a large group of refugees and asylum seekers over the course of one year during the COVID-19 pandemic.

Visa insecurity and family separation have both been shown to be associated with higher levels of mental health difficulties in refugees. Holding an insecure or temporary visa has been linked to increased levels of PTSD [4,13,14], depression [4,8,13,14], and anxiety symptoms [8,13], as well as elevated suicidality [4] and psychosocial difficulties in children and adolescent refugees [15] compared to refugees with permanent or secure visas. While most studies have been cross-sectional, one study has reported that maintaining insecure visa status over 2 years was associated with prolonged PTSD and increases in anxiety and depression [16]. Conversely, there is evidence that transition from temporary to permanent status is linked to reduced psychological symptoms in refugees, including PTSD, depression, as well as alleviated social and immigration-related stressors in different cohorts of refugees and asylum seekers in Australia [6,17]. These studies highlight the detrimental effect of visa insecurity on the mental health of refugees over time.

Similarly, refugees separated from their family report higher PTSD [5,7,18], depression [5,7,18,19], and anxiety symptoms, elevated disability [18], and reduced quality of life [7] compared to refugees not separated from their families. Like research on visa insecurity, most quantitative studies have been cross-sectional with the exception of Fogden et al. [20], who found that worry about absent family members, rather than family separation itself, contributed to the maintenance of PTSD symptoms and an increase in general distress over time. Emerging psychological models of family separation highlight that uncertainty regarding family separation may have a potent effect on mental health because it undermines intrinsic attachment systems [21]. Moreover, separated family members may be under threat while living in insecure contexts, thereby in perpetuating worry and psychological symptoms of displaced refugees [22,23]. There is a need to better understand the temporal relationships between visa insecurity and family separation uncertainties, and mental health outcomes for refugees.

Another salient form of uncertainty that refugees have navigated in recent years is the COVID-19 pandemic and its public health response. Research indicates that COVID-19 related fears were high amongst refugee populations [12,24,25], and were associated with increased PTSD, depression, health anxiety, generalized anxiety, stress, and disability symptoms, particularly when the pandemic reminded refugees of their past trauma [12]. Again, there have been few longitudinal studies to understand the long-term impact of COVID-19 on mental health in refugee cohorts. One study conducted amongst Syrian refugees displaced in Turkey reported that COVID-19 stressors predicted elevated depression and anxiety over 15 months during the pandemic, and that reduced hope predicted elevated stress [26]. Another study conducted amongst displaced Syrian refugees residing in a camp in Jordan found that COVID-19 worries were predicted by greater levels of anxiety during the pandemic, but unexpectedly, lower depression prior to the pandemic [25]. Studies conducted in general populations have also highlighted how contextual factors—including pre-existing social and economic stressors—were important determinants of pandemic-related mental health [27,28,29]. Collectively, these studies highlight the importance of considering broader contextual factors, including different sources of uncertainty, on mental health outcomes during global crises such as the COVID-19 pandemic.

While visa insecurity, family separation, and the COVID-19 pandemic are inherently uncertain external events, an internal factor that may contribute to modulating the psychological impact of these stressors is an individual’s capacity to tolerate uncertainty. Intolerance of uncertainty (IU) is defined as “an individual’s dispositional incapacity to endure the aversive response triggered by the perceived absence of salient, key, or sufficient information, and sustained by the associated perception of uncertainty” [30]. IU is argued to be a cognitive vulnerability that poses a transdiagnostic risk and maintenance factor for PTSD [31,32], anxiety and depression [33]. There are two sub-types of IU: “prospective” IU refers to cognitions that preference predictable events and situations (thereby showing elevated intolerance for uncertain events) and “inhibitory” IU refers to tendencies to engage in avoidant behaviours and functional impairment in the face of uncertainty. While both IU sub-types have been associated with psychopathology overall, there also appears to be evidence of distinct relationships with psychological outcomes. For example, inhibitory IU appears to be particularly associated with PTSD symptoms [34,35], including in refugee populations [36]. Moreover, the context appears important. Models suggest the association between IU and poor mental health may be particularly high in volatile environments and situations that are inherently uncontrollable [37]. This is particularly relevant for refugees. For instance, Nickerson et al. [38] found that negative coping strategies (including IU) were linked to elevated psychological symptoms when refugees were experiencing elevated post-migration stress in a transitory setting. While IU has been identified as a risk factor for fear responses [39] and psychological distress relating to lock-down measures employed to control the spread of COVID-19 [40], no study has considered the role that IU might have in driving psychological symptoms in refugee populations experiencing different forms of uncertainty.

This study aimed to investigate the contributing effects of different forms of uncertainty on changes in mental health symptoms for refugees living in Australia during 12 months at the peak of the COVID-19 pandemic (June 2020–June 2021). We predicted increases in PTSD, depression, and generalized anxiety symptoms over time would be temporally preceeded by higher levels of uncertainty related to visa insecurity, family separation and COVID-19 pandemic (Hypothesis 1). We also hypothesized that the intolerance of uncertainty (IU)—particularly inhibitory IU—would be temporally associated with elevated PTSD, depression and anxiety over time, and contribute to exacerbating visa insecurity, family separation and COVID-19 uncertainties (Hypothesis 2).

## 2. Materials and Methods

### 2.1. Participants

Participants were refugees and asylum seekers who had arrived in Australia between 2011 and 2018, and had participated in the Refugee Adjustment Study [4]. Participants were originally recruited using snowball sampling via advertising at settlement and community services, as well as via social media channels. Criteria for inclusion in this study were identifying as a refugee or asylum seeker having arrived in Australia after January 2011, being >18 years old, and able to complete an online (or paper and pencil) survey in either Arabic, Tamil, Farsi, or English. In June 2020 (approximately 2 months after COVID-19 public health measures were initiated in Australia, including a nation-wide lock-down), Refugee Adjustment Study participants (*N* = 1012) were invited to complete a specific survey designed to measure the acute effects of the COVID-19 pandemic, with a one-month window for completion [12]. A total of *n* = 656 participants responded (65.1% response rate) to form the first timepoint of the current study (T1). In June 2021, a 12 month follow-up survey representing timepoint 2 (T2) was sent to Refugee Adjustment Study participants (*N* = 919), with *n* = 560 participating representing a response rate of 60.9%. At this time, Australia was largely free of COVID-19, but international borders remained closed. Towards the end of the data collection period, there was a rise in COVID-19 cases resulting in widespread national lockdowns, the closure of borders between states, and the policing of communities to comply with public health orders. The final sample comprised *n* = 733 with at least one timepoint of data. Written informed consent was provided in accordance with approval from the UNSW Human Research Ethics Committee (HC180627), and participants were reimbursed with an AUD $25 shopping voucher upon the completion of each survey.

### 2.2. Measures

#### 2.2.1. Demographic Measures

All survey questions were translated and blind back-translated into the three study languages (Arabic, Tamil, Farsi) from English by accredited translators according to World Health Organization protocols, and discrepancies resolved via consultation with bilingual mental health experts. Trauma exposure was measured via the Harvard Trauma Questionnaire (HTQ; [41]) measuring exposure to 16 potentially traumatic events commonly reported by refugee populations (e.g., deprivation, violence, conflict) [42]. The measurement of exposure to potentially traumatic events (PTEs) was based on whether participants reported direct experience and/or witnessing the event, with a count of total event types computed for analysis.

#### 2.2.2. Psychological Symptoms Measures

Post-traumatic stress symptoms were measured via the Post-traumatic Diagnostic Scale for DSM-IV (PDS; [43]) (e.g., “*Having bad dreams or nightmares about the traumatic event*”), adjusted to include additional items to accommodate DSM-5 criteria [4] (e.g., “*Taking lots of risks or doing things that might hurt you*”), with responses provided on a 4-point Likert scale in relation to symptom experiences over the previous month (0 = Not at all or only once; 3 = 5 or more times a week/almost always). The average symptom score was computed across 20 symptom items to indicate PTSD symptom severity. Depression was indexed by the 9-item Patient Health Questionnaire (PHQ-9; [44]), a well-validated brief screening tool for major depression. Participants rated the frequency of symptoms over the previous two weeks (e.g., “*Little interest or pleasure in doing things*”) on a 4-point Likert scale (0 = not at all; 3 = nearly every day). An average score was calculated to reflect depression symptom severity. Anxiety symptoms were measured by the 7-item Generalized Anxiety Disorder (GAD-7; [45]). Participants rated their symptoms over the previous week (e.g., “*Worrying too much about different things*”) on a 4-point Likert scale (1 = not at all; 4 = nearly every day). Items were averaged to reflect anxiety symptom severity. Internal consistency was excellent across PTSD, depression, and anxiety indicators, with Cronbach alpha scores exceeding 0.93 for T1 and T2 measurements.

#### 2.2.3. Visa Status, Family Separation, and COVID-19 Stress Measurement

Visa status and family separation uncertainties were each indexed by three specific items of the Post-Migration Living Difficulties Checklist [46,47] adapted to the Australian context in the Refugee Adjustment Study [4,5]. Participants rated the degree of each difficulty experienced over the previous two months for T1 (to reflect the onset of the COVID-19 pandemic in March–April 2020) and over the previous 12 months for T2 on a 5-point Likert scale (ranging from 1 = was not a problem or did not happen to 5 = a very serious problem). Visa status uncertainty was indexed by three items reflecting key stress points associated with protracted insecure visa status for refugees in Australia: “*Not being allowed to apply for a permanent visa*”, “*Being fearful about being sent to Australian detention centre or an off-shore processing facility* (e.g., *Manus Island, Nauru*)”, and “*Worry that your refugee status or visa status will never be resolved in Australia*”. The mean score was computed as an index visa-status uncertainty, with good internal consistency at T1 (α = 0.86) and T2 (α = 0.87).

Family status uncertainty was also measured by three items of the PMLD Checklist, specifically “*Separation from your family*”, “*Worry about family back home*”, and “*Being unable to return home in an emergency*”. Internal consistency for mean scores of the family-separation uncertainty index was good for T1 (α = 0.81) and T2 (α = 0.82).

COVID-19 stressors were measured in a specifically developed scale administered in a similar manner to the PMLD Check-List, as previously published [12]. The measure comprised 18 items and included stressors relating to infection (e.g., “*Worry about contracting COVID-19*”), public health measures (e.g., “*Having to remain at home*”), accessing services and trusting authorities (e.g., “*Difficulty accessing emergency Government support*”), or fears relating to the pandemic (e.g., “*Being reminded of difficult or stressful experiences from the past*”). Participants indicated whether each stressor had been a problem for them on a 5-point Likert scale ranging (1 = was not a problem or did not happen; 5 = a very serious problem). Responses on the 18 items were averaged to indicate the severity of COVID-19 stressors, and internal consistency was strong at T1 (α = 0.93) and T2 (α = 0.94).

#### 2.2.4. Intolerance of Uncertainty (IU)

IU was indexed by the Intolerance of Uncertainty short form measure [48]. Of the 12 items, seven items indexed prospective IU (e.g., “*Unforeseen events upset me greatly*”), and five items measured inhibitory IU (e.g., “*When I am uncertain, I can’t function very well*”). Responses were provided on a 5-point Likert scale (ranging from 1 = not at all like me to 5 = extremely like me) that reflect dispositional IU traits. Mean scores of each subscale were computed such that higher scores reflected a stronger IU on prospective and inhibitory dimensions. Internal consistency was good for both time points (prospective IU: T1 α = 0.85; T2 α = 0.87; inhibitory IU α = 0.89; T2 α = 0.90).

### 2.3. Data Analysis

**Figure 1:**
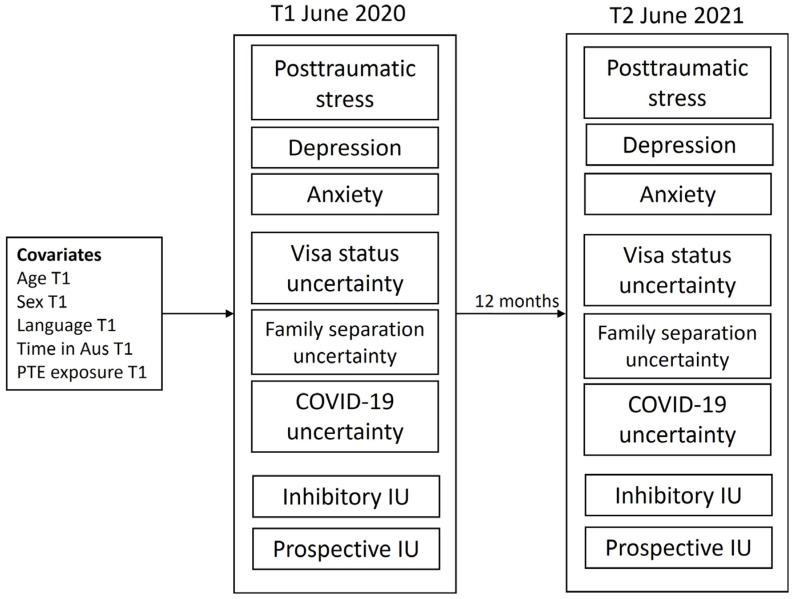
Cross-lagged panel model tested across T1 and T2 including covariates. PTE—potentially traumatic event count; IU—intolerance of uncertainty.

A cross-lagged panel model analysis was used to investigate the temporal relationship between PTSD, depression, and anxiety symptoms; visa status, family separation and COVID-19-related stressors, and prospective and inhibitory intolerance of uncertainty at timepoint 1 (T1) to 12 months later to timepoint 2 (T2). The full maximum likelihood methods were used to account for missing data at T2. A cross-lagged panel model was specified whereby PTSD, depression, anxiety, visa status, family separation, and COVID-19 uncertainties, and prospective and inhibitory IU at T1 predicted these same measures at T2. Accordingly, the model comprised auto-regressive paths (i.e., where one variable predicted the same variable 12 months later, for example, PTSD at T1 predicted PTSD at T2) and cross-lagged paths (i.e., where variables at T1 predicted all other variables at T2). This approach allows for testing temporal ordering by determining whether changes in one variable predict changes in another variable over and above the baseline levels of the first variable. For example, the presence of a significant positive cross-lagged path between visa uncertainty at T1 and PTSD at T2 in the presence of an autoregressive path between PTSD at T1 and PTSD at T2 would suggest that visa uncertainty was associated with subsequent increases in PTSD. Cross-lagged paths were used to test hypothesis 1 (e.g., that contextual uncertainty would be associated with subsequent increases in psychological symptoms) and hypothesis 2 (e.g., that intolerance of uncertainty would be associated with subsequent increases in psychological symptoms and uncertainty stress). A representation of the cross-lagged panel model tested is provided in Figure 1.

All T1 variables were allowed to correlate, and all T2 variables were allowed to correlate. Age, gender, time in Australia, lifetime PTE exposure, and language group were included as covariates at T1. These factors are known to influence psychological symptom severity and stressor ratings amongst refugee populations [5,6,49], and we sought to control for their influence in the model. Language group was dummy-coded, with English being the reference group. Goodness-of-model fit was determined using the comparative fit index (CFI > 0.95), Tucker–Lewis index (TLI > 0.95), root mean square error of approximation (RMSEA < 0.05), and standardized root mean squared residual (SRMR < 0.05). Pathways were deemed significant at *p* < 0.05. All analyses were conducted in MPlus version 8.8 [50]. Figure 1 presents the full model tested.

## 3. Results

### 3.1. Participant Characteristics

Participant characteristics are provided in Table 1.

There were no significant differences between T1 and T2 samples on demographic factors. Half of the sample at both timepoints were female, and the mean age of the samples was 42–43 years. Most participants completed the study in Arabic, with the largest proportion of participants originating from Iraq, followed by Syria, and participants had experienced a mean of 3.88 pre-migration PTEs (noting trauma exposure was measured once). We tested the differences between participants who were retained across both time points compared to those lost to T2 on time 1 variables, and found only a significant difference for time in Australia (those who were retained had spent less time living in Australia (F(1,726) = 10.003, *p* = 0.002). We also compared participants who completed both timepoints (n = 483) compared to those who only completed T1 (n = 250), and found a significant difference only for prospective IU, whereby participants who completed both timepoints reported higher prospective IU compared to those who completed only one timepoint (F(1,609) = 10.95, *p* < 0.001). Therefore, we concluded that there was little difference between T1 and T2 cohorts on key variables in the model.

### 3.2. Cross-Lag Panel Analysis

Due to missing data on covariate information (predominantly PTE exposure) representing 9% of the sample, the final sample used in the analysis comprised n = 649. Correlations between all variables at both timepoints are provided in Supplementary Results Table. The model showed good fit to the data: CFI = 0.99, TLI = 0.98, RMSEA = 0.03 (95% C.I. 0.02–0.04), SRMR = 0.02. All auto-regressive paths were significant (*p* < 0.05).

**Figure 2:**
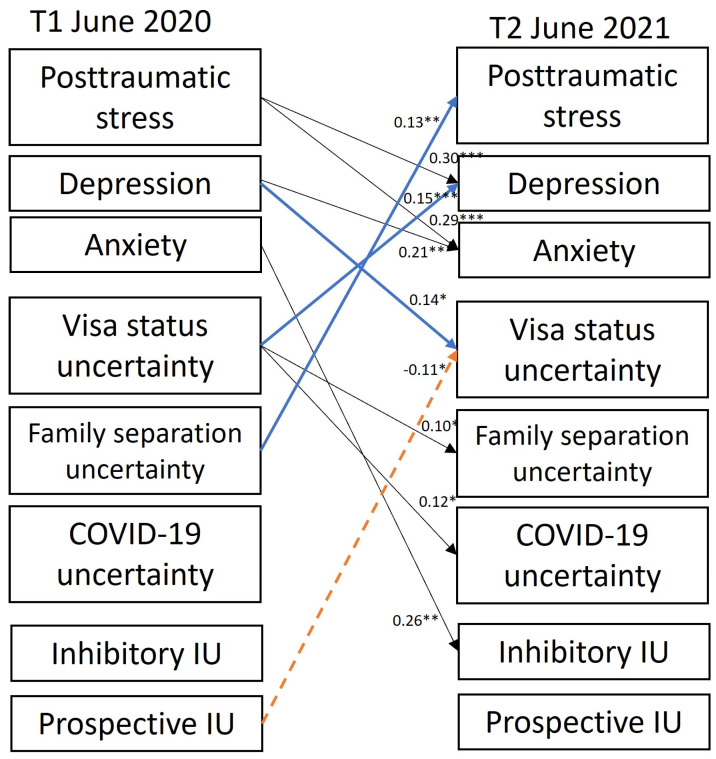
Significant cross-lagged pathways between variables measured at T1 and T2. Solid arrows refer to positive associations and dashed arrows refer to negative associations. Pathways presented in blue are significant cross-lagged pathways under hypothesis 1; pathways in orange are significant cross-lagged pathways under hypothesis 2; black pathways are additional significant cross-lagged pathways observed between different psychological outcomes, sources of uncertainty and intolerance of uncertainty (IU) subtypes. Standardized beta weights (β) are provided on significant cross-lagged pathways; * *p* < 0.05, ** *p* < 0.01, *** *p* < 0.001. See Table 2 for specific data.

Significant cross-lagged paths are depicted in Figure 2 and all pathways are reported in Table 2 (covariate pathways are reported in Supplementary Results). In support of hypothesis 1 (blue pathways, Figure 2), higher levels of visa-status uncertainty at T1 were associated with significant increases in depression at T2, while a higher depression at T1 was associated with significant increases in visa status uncertainty at T2. This indicates a bidirectional temporal effect between visa status uncertainty and depression symptoms. Family separation uncertainty at T1 was specifically related to increases in PTSD symptoms at T2. COVID-19 uncertainty was not significantly associated with any psychological symptoms in either direction, although visa status uncertainty at T1 notably predicted increases in both COVID-19 and family separation uncertainty at T2.

In contrast to hypothesis 2, prospective IU at T1 was associated with significantly less visa uncertainty at T2 (broken orange pathway in Figure 2). The only other significant cross-lag pathway involving IU was that higher anxiety symptoms at T1 were associated with increases in inhibitory IU at T2. Cross-lagged pathways between COVID-19 uncertainty, family separation uncertainty, inhibitory IU, and T2 psychological outcomes were non-significant.

Other significant cross-lagged pathways observed (shown in black, Figure 2) were that more severe PTSD symptoms at T1 were associated with increases in depression and anxiety symptoms at T2. Higher depression at T1 was associated with increases in GAD symptoms at T2.

Covariate pathways are provided in Supplementary Results. In brief, age was negatively associated with visa uncertainty at T1, COVID-19 uncertainty at T1, and inhibitory IU at T1. Female sex was associated with a higher PTSD, depression, generalized anxiety symptoms at T1. Longer time in Australia was positively associated with all variables except COVID-19 uncertainty and prospective IU. PTE exposure was positively associated with all variables in the model. Completing the survey in Farsi was associated with greater visa status uncertainty, family separation uncertainty, and COVID-19 uncertainty at T1. Completing the surveys in Tamil was associated with higher levels of visa status uncertainty at T1.

## 4. Discussion

This study examined how different sources of refugee uncertainty—namely visa status, family separation, and the COVID-19 pandemic, shaped psychological symptoms in a cohort of refugees and asylum-seekers settled in Australia over 12 months during the height of the COVID-19 pandemic. Overall, we found partial support for our hypotheses. Findings revealed that different forms of refugee uncertainty have specific impacts on psychopathology over time. We observed a bidirectional relationship between visa status uncertainty and depression between T1 and T2, such that visa status uncertainty contributed to increases in depression over time, and that depression contributed to increases in visa status uncertainties over time. Family separation uncertainty at T1 was specifically associated with elevated PTSD symptoms at T2. Counter to hypothesis 1, COVID-19 related uncertainty was not associated with change in psychological symptoms, but rather visa-status uncertainty at T1 was linked to increases in COVID-19 uncertainty at T2. Hypothesis 2 was largely unsupported. IU featured in two cross-lagged relationships in the model, where GAD symptoms at T1 were temporally associated with inhibitory IU scores at T2 and prospective IU at T1 predicted lower levels of visa-status uncertainty at T2. This finding will be unpacked below. Overall, this model highlights the corrosive impact of various forms of refugee uncertainty on specific psychological symptoms, which persist in the face of another emergency represented by the COVID-19 pandemic.

The study found specific relationships between sources of uncertainty and psychological symptoms. First, the specificity of the bidirectional link between visa-status related uncertainty and depression aligns with the “lethal hopelessness” psychological profile that reflects the despair reported by refugees and asylum seekers experiencing a protracted visa status resolution process [51]. Lethal hopelessness is marked by elevations in self-harm and suicidal behaviours [51]. Prolonged visa status uncertainty limits capacity for full settlement and self-determination, which may have a biological basis in how core brain networks important for self-representation communicate with one another [52]. When insecure visa status is resolved to permanency, previous research has demonstrated an alleviation of depression symptoms [6,17], although these longitudinal studies also observed a parallel reduction in PTSD symptoms. Outside of our hypotheses, we also observed that visa status uncertainty at T1 was linked to increases in both COVID-19-related stress and family separation uncertainty at T2. Worries related to visa insecurity may temporally precede a diminished capacity to cope with COVID-19, potentially increasing the burden of the pandemic on refugees experiencing higher visa status uncertainty [9,10,11].

Family separation uncertainty at T1 was uniquely associated with increased levels of PTSD symptoms over 12 months of the pandemic. This finding builds on the established cross-sectional link between family separation and PTSD [5,7,53], and the observation that worry about separated family contributed to elevated PTSD symptoms over time [20]. The connection between family separation and PTSD accords with an emerging conceptualization of refugee family separation as a form of ongoing trauma and systemic violence [7,22]. Family separation can pose an increased risk to the resettled refugee due to constant worry about absent family living in insecure settings, facing persecution, conflict, or forced displacement [20,22]. The absence of a family member may result in increased social stress [5] or reduced buffering against social and other forms of stress [21,22]. Worry about separated family members may have been exacerbated during the COVID-19 pandemic, who may have been living in COVID ‘hot-spot’ countries or in other high-risk settings where public health measures to curb the threat of COVID-19 were limited (e.g., in refugee camps, immigration detention) [12]. Alternatively, attachment anxieties are increased during times of stress—such as that represented by the pandemic—and this in itself may have elevated concerns about separation from others [54]. Overall, the current finding highlights ongoing separation as a key issue that may impair trauma recovery processes in displaced populations.

The capacity to tolerate uncertain situations was not specifically linked to psychological outcomes over time in the model, although anxiety symptoms at T1 were associated with increases in inhibitory IU at T2. This reflects a reversal of findings reported in other refugee populations, where inhibitory IU has been associated with increases in PTSD symptoms over time [36]. This may be explained by the construction of the current model, which considered contextual stressors alongside psychological symptoms. Moreover, the intolerance of uncertainty short-form (IU-12) measures trait IU [48]; if it measured state IU in relation to the COVID-19 pandemic [55], stronger associations with psychological symptoms may have been observed. For instance, specifically measuring IU in relation to the measures implemented to curb the effects of COVID-19 (e.g., lock-downs, restriction on international travel, work from home, online learning), may have changed the statistical association between IU inhibitory and mental health. An unexpected finding was that prospective IU was negatively associated with visa status uncertainty, indicating that the greater prospective intolerance of uncertainty was associated with subsequent decreases in visa status uncertainty. The prospective IU items focus on preferences for wanting to know as much about the future as possible, and behavioural tendencies towards minimizing unexpected events [48]. The specific nature of refugee visa insecurity, which involves living in a state of protracted limbo and uncertainty about whether one can live permanently in Australia, may explain why higher prospective IU was associated with lower levels of visa-status uncertainty. It is possible that refugees with insecure visas have higher learned helplessness [56], alongside “lethal helplessness” [51], which involves accepting that any actions have no impact on future outcomes. The role of context may also be critical to consider when interpreting these findings. Research conducted with first responders (e.g., firefighters) found that the intolerance of uncertainty was lower compared to other clinical and community samples, regardless of psychological disorder diagnosis, suggesting that repeated exposure to uncertain events changes the relationship between IU and psychological risk [57]. A similar adaptation may occur for refugee populations exposed to multiple traumatic events and stressors, although our model only observed such a pattern for visa status insecurity. Moreover, there may be cultural variations in appraisals relating to the intolerance of uncertainty that are not factored into this study. Nations vary at the group-level along a dimension of uncertainty avoidance to acceptance [58]. Our sample included refugee participants from a range of countries of origin, which may have affected findings. However, the measurement at the individual level should still capture broader cultural influences. Additionally, there is evidence that nationality does not moderate the relationship between IU and depression, but does affect how IU relates to measures of mental wellbeing [59]. Generally, the relationship between IU and refugee mental health is an under-researched area and will need to be unpacked further in future research.

These findings highlight the excessive level of uncertainty that refugees live with, and the specific impact of these different sources of uncertainty on psychological health. Policies and programs that work to reduce levels of uncertainty are potentially key to improving psychological wellbeing in refugee populations. From an intervention perspective, targeting the uncertainty experienced by refugees may be a useful component of therapy as a transdiagnostic mechanism, and provide skills to manage situations that cannot be changed. These findings could also have broader implications for responding to the needs of refugee communities during future major crises such as another global pandemic, natural disasters, or other emergency humanitarian events.

This study highlights the variable mental health experiences of refugees according to their contexts and circumstances, pointing to the inadequacies of a one-sized-fits-all approach to responding to mental health needs—particularly in a public health emergency. Therefore, it is critical that public health mitigation or disaster response programs recognize how refugees may specifically respond to the uncertainties posed by these large-scale events, given ongoing uncertainties over the visa and family status experienced by many refugees. Furthermore, refugee populations may have psychological health concerns that may be exacerbated by emergency situations. For instance, family separation that was prolonged due to border closures and restricted reunification pathways appear to elevate post-traumatic stress symptoms in this study. Mental health interventions could be adapted so that intrusion, hyperarousal, and avoidance symptoms associated with fear related to separation from family can be explicitly addressed in treatment [60].

The socioecological model of refugee mental health recognizes how critical the social context is to refugee mental health, a model that is supported by the findings of this study [61]. The evidence base for how to target interventions specifically to address these stressors is still limited. Community-based responses may be an essential component to supporting refugee mental health during times of crisis. For example, social losses due to ongoing family separation could be addressed via psychosocial programs that build community social connections [62]. During the COVID-19 pandemic, however, public health priorities in Australia paused community programs and prevented access to this vital avenue of support, which was associated with poorer mental health [12]. At the same time, despite evidence of uneven digital access and digital literacy among refugee populations (i.e., lower use in women and older people), the use of digital technology by refugees during COVID-19 provided a lifeline to maintain social connections within Australia and with friends and families overseas [63]. Future strategies should incorporate contingencies, including digital technology, to enable refugees to continue to engage in important social activities. Given evidence of refugees’ increased use of digital technology during COVID-19 [63], targeted digital interventions may offer a way forward that fosters community and social connections to support mental health, including distress related to family separation [64], but may also carry additional risks including discrimination, cyber-bullying, data security, increased surveillance, and increased marginalization [65,66]. Additionally, psychological interventions that focus on stress management, including low intensity strategies that can be rapidly scaled up, could be important tools to respond to the mental health needs of refugees in crises like pandemics. However, it is vital to note that the evidence base for the effectiveness of scalable interventions for refugee populations remains mixed [67].

More generally, from a systems perspective, it is recommended that public health responses are adapted to accommodate help-seeking and ongoing access to psychological care for refugees. Government authorities could maintain transparent communication regarding refugee status determination, visa application, and family status processes to reduce the uncertainties associated with these processes. Critically, the findings indicate that, to mitigate psychological risks as well as facilitate better stress responsivity and health and safety outcomes for refugees, permanent settlement solutions and prioritizing family reunification should be an important component of a complex emergency response mechanism for nations hosting and resettling refugees.

The current findings must be interpreted considering the study limitations. While the study is longitudinal, the model does not account for pre-pandemic levels of psychopathology which may play an important role in shaping the pathways observed. We modelled broad COVID-19 stressors to create the most parsimonious model but there may be specific COVID-19 stressors that more strongly affect mental health outcomes over time [12]. While the sample is not representative of refugee arrivals in Australia between 2011 and 2018 due to the convenience and snowball sampling methodology, the cohort does reflect the key language groups and original countries of humanitarian arrivals and asylum seekers during this time [4]. While the intolerance of uncertainty was measured in this study, we did not observe a significant relationship with mental health symptoms, contrary to other studies conducted with the refugee [36] and non-refugee samples [31,32,33]. Future studies could examine this in further detail, and explore cultural variations in the perceptions and understandings of tolerance of uncertainty. Moreover, exploring the potential moderating influence of IU on the temporal relationships between forms of uncertainty and mental health outcomes could be explored in future investigations. Further studies could also examine the role of other psychological coping mechanisms—including emotion regulation [49] and self-efficacy [68]—both known to be important factors associated with refugee wellbeing during stress. The findings may be specific to the Australian context. Looking beyond Australia to other host nations to understand more broadly the mental health impact of different forms of uncertainties in different contexts would be useful. Finally, this study examined two timepoints that were 12 months apart. Subsequently, studies extending this timeframe would be imperative to examine the lasting effects of uncertainty following the COVID-19 pandemic on refugees.

## 5. Conclusions

This study revealed that, while psychological symptoms worsened in a large cohort of refugees in Australia during the COVID-19 pandemic, uncertainty related to visa status and family separation had specific associations with psychopathology over time. Understanding the complex needs of refugees and asylum seekers during a global pandemic is critical to ensure that response programs and services are oriented to best support refugee communities in future emergencies.

## Figures and Tables

**Table 1:** Participant characteristics.

		Timepoint 1 (T1) June 2020 N = 656		Timepoint 2 (T2) June 2021 N = 560	
		N/Mean	%/SD	N/Mean	%/SD
Age (years)		42.83	12.32	43.68	12.53
Sex	Male	333	50.8%	278	49.6%
	Female	323	49.2%	282	50.4%
Language	Arabic	514	78.4%	411	73.4%
	Farsi	65	9.9%	45	8.0%
	English	59	9.0%	48	8.6%
	Tamil	18	2.7%	56	10%
Country of origin	Iraq	406	61.9%	356	63.6%
	Syria	118	18.0%	82	14.6%
	Iran	67	10.2%	62	11.1%
	Sri Lanka	20	3.0%	23	4.1%
	Afghanistan	10	1.5%	10	1.8%
	Other	35	5.3%	27	4.8%
Marital status	Married/partnered	503	76.7%	424	75.7%
	Widow/widower	17	2.6%	19	3.4%
	Divorced/separated	21	3.2%	20	3.6%
	Single/never married	113	17.2%	95	17.0%
	Unknown	2	0.3%	2	0.4%
Visa status	Secure visa	541	82.5%	451	80.5%
	Insecure visa	110	16.8%	102	18.2%
	Unknown	5	0.8%	6	1.1%
Time in Australia (years)		4.60	3.81	5.71	1.71
PTE exposure (experienced/witnessed)		3.88	4.08	-	-
Post-traumatic stress symptoms (PDS; DSM-5); mean		0.61	0.70	0.55	0.66
Depression symptoms (PHQ-9); mean		0.81	0.72	0.81	0.74
Anxiety symptoms (GAD-7); mean		0.85	0.73	0.76	0.76
Visa status uncertainty stressors; mean		1.59	1.09	1.65	1.13
Family separation uncertainty stressors; mean		2.36	1.25	2.44	1.28
COVID-19 uncertainty stressors; mean		2.05	0.78	2.05	0.83
Inhibitory IU; mean		2.54	0.87	2.44	0.91
Prospective IU; mean		2.80	0.77	2.71	0.84

**Table 2:** Autoregressive paths and cross-lagged paths. Significant paths *p* < 0.05. Arrows refer to direction of effects. Acronyms: PTSD—post-traumatic stress disorder; IU—intolerance of uncertainty.

		B (s.e)	β	*t*	*p*
Autoregressive Paths					
T1 PTSD symptoms →	T2 PTSD symptoms	0.62 (0.06)	0.69	10.06	<0.001
T1 depression symptoms →	T2 depression symptoms	0.39 (0.07)	0.39	6.02	<0.001
T1 anxiety symptoms →	T2 anxiety symptoms	0.29 (0.07)	0.29	4.22	<0.001
T1 visa uncertainty →	T2 visa uncertainty	0.78 (0.04)	0.77	21.39	<0.001
T1 family separation uncertainty →	T2 family separation uncertainty	0.67 (0.04)	0.69	15.75	<0.001
T1 COVID-19 uncertainty →	T2 COVID-19 uncertainty	0.54 (0.05)	0.53	10.26	<0.001
T1 inhibitory IU →	T2 inhibitory IU	0.31 (0.07)	0.30	4.60	<0.001
T1 prospective IU →	T2 prospective IU	0.34 (0.07)	0.32	5.18	<0.001
Cross-lagged paths					
T1 PTSD symptoms →	T2 depression symptoms	0.31 (0.07)	0.30	4.68	<0.001
	T2 anxiety symptoms	0.30 (0.07)	0.29	4.27	<0.001
	T2 visa uncertainty	0.02 (0.01)	0.01	0.20	0.844
	T2 family separation uncertainty	−0.09 (0.12)	−0.05	−0.77	0.442
	T2 COVID-19 uncertainty	0.05 (0.10)	0.04	0.53	0.597
	T2 inhibitory IU	0.05 (0.12)	0.04	0.43	0.667
	T2 prospective IU	0.09 (0.10)	0.08	0.91	0.363
T1 depression symptoms →	T2 PTSD symptoms	0.08 (0.06)	0.01	1.37	0.172
	T2 anxiety symptoms	0.21 (0.07)	0.21	3.03	0.002
	T2 visa uncertainty	0.22 (0.01)	0.14	2.25	0.025
	T2 family separation uncertainty	0.16 (0.12)	0.09	1.33	0.183
	T2 COVID-19 uncertainty	0.13 (0.10)	0.12	1.40	0.162
	T2 inhibitory IU	0.09 (0.12)	0.08	0.86	0.389
	T2 prospective IU	0.04 (0.10)	0.04	0.432	0.665
T1 anxiety symptoms →	T2 PTSD symptoms	−0.04 (0.06)	−0.05	−0.65	0.518
	T2 depression symptoms	0.05 (0.06)	0.05	0.70	0.482
	T2 visa uncertainty	−0.04 (0.10)	−0.03	−0.40	0.688
	T2 family separation uncertainty	0.07 (0.11)	0.04	0.63	0.527
	T2 COVID-19 uncertainty	0.01 (0.09)	0.004	0.05	0.957
	T2 inhibitory IU	0.31 (0.10)	0.26	3.01	0.003
	T2 prospective IU	0.18 (0.10)	0.16	1.83	0.068
T1 visa uncertainty →	T2 PTSD symptoms	−0.02 (0.02)	−0.03	−0.82	0.414
	T2 depression symptoms	0.10 (0.03)	0.15	3.97	<0.001
	T2 anxiety symptoms	0.03 (0.03)	0.05	1.17	0.243
	T2 family separation uncertainty	0.11 (0.05)	0.10	2.40	0.016
	T2 COVID-19 uncertainty	0.09 (0.03)	0.12	2.56	0.010
	T2 inhibitory IU	0.04 (0.04)	0.05	0.98	0.328
	T2 prospective IU	0.02 (0.04)	0.03	0.49	0.624
T1 family separation uncertainty →	T2 PTSD symptoms	0.07 (0.02)	0.13	2.76	0.006
	T2 depression symptoms	0.04 (0.03)	0.07	1.53	0.126
	T2 anxiety symptoms	0.002 (0.03)	0.003	0.06	0.952
	T2 visa uncertainty	−0.01 (0.04)	−0.01	−0.20	0.844
	T2 COVID-19 uncertainty	−0.01 (0.03)	−0.01	−0.24	0.810
	T2 inhibitory IU	−0.05 (0.04)	−0.07	−1.15	0.251
	T2 prospective IU	0.02 (0.04)	0.03	0.57	0.569
T1 COVID-19 uncertainty →	T2 PTSD symptoms	−0.03 (0.04)	−0.04	−0.75	0.454
	T2 depression symptoms	−0.05 (0.04)	−0.05	−1.13	0.258
	T2 anxiety symptoms	−0.05 (0.04)	−0.06	−1.23	0.217
	T2 visa uncertainty	0.01 (0.06)	0.01	0.123	0.902
	T2 family separation uncertainty	−0.06 (0.07)	−0.04	−0.86	0.392
	T2 inhibitory IU	−0.08 (0.06)	−0.08	−1.31	0.189
	T2 prospective IU	−0.05 (0.06)	−0.04	−0.76	0.448
T1 inhibitory IU →	T2 PTSD symptoms	0.03 (0.04)	0.04	0.78	0.438
	T2 depression symptoms	0.06 (0.04)	0.07	1.31	0.191
	T2 anxiety symptoms	0.05 (0.05)	0.06	1.19	0.232
	T2 visa uncertainty	0.03 (0.06)	0.02	0.46	0.647
	T2 family separation uncertainty	0.03 (0.08)	0.02	0.41	0.680
	T2 COVID-19 uncertainty	−0.01 (0.06)	−0.01	−0.15	0.880
	T2 prospective IU	0.05 (0.06)	0.06	0.83	0.404
T1 prospective IU →	T2 PTSD symptoms	−0.07 (0.04)	−0.08	−1.68	0.093
	T2 depression symptoms	−0.07(0.04)	−0.08	−1.64	0.100
	T2 anxiety symptoms	−0.002 (0.05)	−0.002	−0.04	0.966
	T2 visa uncertainty	−0.16 (0.07)	−0.11	−2.52	0.012
	T2 family separation uncertainty	−0.05 (0.07)	−0.03	−0.69	0.490
	T2 COVID-19 uncertainty	−0.02 (0.06)	−0.02	−0.40	0.690
	T2 inhibitory IU	0.09 (0.07)	0.08	1.27	0.205

## Data Availability

The data that support the findings of this study are available upon request from the corresponding author, B.L. The data are not publicly available due to the de-identified data possibly containing information that could compromise the privacy and safety of the research participants.

## Supplementary Materials

The following supporting information can be downloaded at https://www.mdpi.com/article/10.3390/ijerph22060855/s1, Table S1: Covariate pathways on T1 variables.

## Abbreviations

The following abbreviations are used in this manuscript:

DSM	Diagnostic and Statistical Manual
GAD	Generalized anxiety disorder
IU	Intolerance of uncertainty
PTS	Post-traumatic stress
PTSD	Post-traumatic stress disorder
T1	Time 1
T2	Time 2
