# Supplementary material for: Longitudinal Associations Between Sources of Uncertainty and Mental Health Amongst Resettled Refugees During the COVID-19 Pandemic
Source: Int J Environ Res Public Health. 2025 May 30;22(6):855. doi: 10.3390/ijerph22060855 (PMC12192629; doi:10.3390/ijerph22060855)
Supplement: Supplementary file 1 [file ijerph-22-00855-s001.zip › ijerph-3555361-supplementary.pdf]

Supplementary Results: Covariate pathways on T1 variables					
		B (s.e)	β	*t*	*p*
Age →	T1 PTSD	-0.001 (0.002)	-0.01	-0.32	0.752
	T1 Depression	-0.001 (0.002)	-0.02	-0.66	0.513
	T1 GAD	-0.002 (0.002)	-0.03	-0.91	0.365
	T1 Visa uncertainty	-0.01 (0.003)	-0.07	-2.04	0.041
	T1 Family separation uncertainty	-0.002 (0.004)	-0.02	-0.65	0.516
	T1 COVID-19 uncertainty	-0.01 (0.003)	-0.10	-2.56	0.011
	T1 inhibitory IU	0.01 (0.003)	0.12	3.03	0.002
	T1 prospective IU	0.004 (0.003)	0.07	1.65	0.099
Gender →	T1 PTSD	0.17 (0.05)	0.12	3.34	0.001
	T1 Depression	0.13 (0.06)	0.08	2.62	0.024
	T1 GAD	0.16 (0.06)	0.11	2.76	0.006
	T1 Visa uncertainty	-0.13 (0.08)	-0.06	-1.70	0.088
	T1 Family separation uncertainty	0.09 (0.09)	0.04	1.07	0.286
	T1 COVID-19 uncertainty	0.03 (0.06)	0.02	0.52	0.602
	T1 inhibitory IU	0.08 (0.07)	0.04	1.06	0.290
	T1 prospective IU	0.00 (0.07)	0.00	0.00	1.000
Time in Australia →	T1 PTSD	0.08 (0.02)	0.20	4.41	<.001
	T1 Depression	0.08 (0.02)	0.18	3.83	<.001
	T1 GAD	0.07 (0.02)	0.16	3.22	0.001
	T1 Visa uncertainty	0.20 (0.03)	0.31	7.52	<.001
	T1 Family separation uncertainty	0.15 (0.03)	0.21	4.94	<.001
	T1 COVID-19 uncertainty	0.04 (0.02)	0.10	1.95	0.051
	T1 inhibitory IU	0.06 (0.03)	0.11	2.09	0.037
	T1 prospective IU	0.01 (0.03)	0.02	0.40	0.690
Potentially traumatic event (PTE) exposure →	T1 PTSD	0.07 (0.01)	0.41	10.64	<.001
	T1 Depression	0.06 (0.01)	0.35	8.92	<.001
	T1 GAD	0.06 (0.01)	0.35	8.65	0.001
	T1 Visa uncertainty	0.07 (0.01)	0.25	7.21	<.001
	T1 Family separation uncertainty	0.12 (0.01)	0.40	11.26	<.001
	T1 COVID-19 uncertainty	0.07 (0.01)	0.36	8.63	<.001
	T1 inhibitory IU	0.05 (0.01)	0.22	4.89	<.001
	T1 prospective IU	0.03 (0.01)	0.16	3.46	0.001
Arabic language →	T1 PTSD	-0.08 (0.10)	-0.05	-0.81	0.418
	T1 Depression	0.01 (0.10)	0.01	0.90	0.928
	T1 GAD	-0.05 (0.12)	-0.03	-0.44	0.662
	T1 Visa uncertainty	0.21 (0.14)	0.08	1.53	0.125
	T1 Family separation uncertainty	-0.30 (0.16)	-0.10	-1.88	0.060
	T1 COVID-19 uncertainty	0.13 (0.11)	0.07	1.19	0.235
	T1 inhibitory IU	-0.03 (0.13)	-0.14	-0.21	0.833
	T1 prospective IU	-0.05 (0.12)	-0.03	-0.39	0.699
Farsi language →	T1 PTSD	-0.10 (0.12)	-0.04	-0.854	0.393
	T1 Depression	0.17 (0.13)	0.07	1.33	0.184
	T1 GAD	-0.13 (0.13)	-0.05	-0.97	0.331
	T1 Visa uncertainty	1.08 (0.17)	0.29	6.30	<.001
	T1 Family separation uncertainty	0.46 (0.20)	0.11	2.37	0.018
	T1 COVID-19 uncertainty	0.36 (0.14)	0.13	2.55	0.011
	T1 inhibitory IU	-0.21 (0.17)	-0.07	-1.24	0.216
	T1 prospective IU	-0.14 (0.15)	-0.05	-0.94	0.349
Tamil language →	T1 PTSD	-0.27 (0.14)	-0.10	-1.92	0.055
	T1 Depression	0.05 (0.16)	0.02	0.301	0.763
	T1 GAD	-0.14 (0.16)	-0.05	-0.83	0.405
	T1 Visa status uncertainty	0.53 (0.20)	0.13	2.66	0.008
	T1 Family separation uncertainty	0.20 (0.24)	0.04	0.86	0.389
	T1 COVID-19 uncertainty	0.04 (0.18)	0.01	0.22	0.835
	T1 inhibitory IU	-0.19 (0.22)	-0.06	-1.24	0.216
	T1 prospective IU	-0.17 (0.21)	-0.06	-.82	0.411
